# Cardiovascular safety with linagliptin in patients with type 2 diabetes mellitus: a pre-specified, prospective, and adjudicated meta-analysis of a phase 3 programme

**DOI:** 10.1186/1475-2840-11-3

**Published:** 2012-01-10

**Authors:** Odd Erik Johansen, Dietmar Neubacher, Maximilian von Eynatten, Sanjay Patel, Hans-Juergen Woerle

**Affiliations:** 1 Boehringer Ingelheim, Asker, Norway; 2 Boehringer Ingelheim Pharma GmbH and Co. KG, Biberach, Germany; 3 Boehringer Ingelheim GmbH, Ingelheim, Germany; 4 Boehringer Ingelheim Ltd, Bracknell, Berkshire, UK

**Keywords:** Cardiovascular risk, DPP-4 inhibitor, linagliptin, meta-analysis, type 2 diabetes mellitus

## Abstract

**Background:**

This study investigated the cardiovascular (CV) safety profile of the dipeptidyl peptidase (DPP)-4 inhibitor linagliptin versus comparator treatments.

**Methods:**

This was a pre-specified meta-analysis of CV events in linagliptin or comparator-treated patients with type 2 diabetes mellitus (T2DM) from eight Phase 3 studies. All suspected CV events were prospectively adjudicated by a blinded independent expert committee. The primary endpoint was a composite of CV death, stroke, myocardial infarction, and hospitalization for unstable angina. Three secondary composite endpoints derived from the adjudicated CV events were also pre-specified. Risk estimates were calculated using several statistical methods including Cox regression analysis.

**Results:**

Of 5239 treated patients (mean ± SD HbA1c 65 ± 10 mmol/mol [8.0 ± 0.9%], age 58 ± 10 years, BMI 29 ± 5 kg/m^2^), 3319 received linagliptin once daily (5 mg, 3159; 10 mg, 160) and 1920 received comparators (placebo, 977; glimepiride 1-4 mg, 781; voglibose 0.6 mg, 162). Cumulative exposure (patient-years) was 2060 for linagliptin and 1372 for comparators. Primary CV events occurred in 11 (0.3%) patients receiving linagliptin and 23 (1.2%) receiving comparators. The hazard ratio (HR) for the primary endpoint showed significantly lower risk with linagliptin than comparators (HR 0.34 [95% confidence interval (CI) 0.16-0.70]) as did estimates for all secondary endpoints (HR ranging from 0.34 to 0.55 [all upper 95% CIs < 1.0]).

**Conclusions:**

These results from a large Phase 3 programme support the hypothesis that linagliptin may have CV benefits in patients with T2DM.

## Background

Despite continuing medical and pharmacological efforts, patients with type 2 diabetes mellitus (T2DM) still bear a substantial burden of increased cardiovascular (CV) morbidity and premature mortality [1,2]. Although many risk factors are involved, hyperglycaemia remains an important contributor to increased CV disease incidence and seems to potentiate the deleterious effects of lipids and blood pressure elevation [2,3]. Nonetheless, recent large outcomes trials of glycaemic intervention in general and of intensive treatment in particular have shown conflicting results in terms of CV benefits for patients with T2DM [4-7]. The ambivalence of these findings has led to development of the hypothesis that the effectiveness of intensive glucose control likely depends on individualizing treatment (e.g. treatment modality and glycaemic target) to account for CV risk and other factors [8]. In particular, weight gain and increased hypoglycaemia are often associated with established glucose-lowering treatments that increase insulin secretion (in a glucose-independent manner) or insulin sensitivity and may heighten CV risk. Some agents, such as rosiglitazone, have been shown to increase risk for CV events possibly due to unanticipated pleiotropic CV effects [9]. In light of these concerns, regulatory authorities, including the US Food and Drug Administration (FDA) and the European Medicines Agency, have issued guidance that the development programmes for all new glucose-lowering therapies must show that treatment confers no unacceptable increases in CV risk [10,11].

The need to improve glycaemic control while minimizing harmful side effects has led to interest in therapeutic approaches aimed at avoiding such pitfalls. Dipeptidyl peptidase (DPP)-4 inhibitors, which enhance postprandial levels of the incretin hormones glucagon-like peptide (GLP)-1 and glucose-dependent insulinotropic polypeptide (GIP), have limited side effects [12]. The glucoregulatory actions of incretins include glucose-dependent promotion of insulin secretion, glucagon suppression, delayed gastric emptying, and increased satiety.

Linagliptin is a DPP-4 inhibitor that was recently approved as a once-daily oral glucose-lowering drug in the USA, Japan, and Europe. Its molecular structure is xanthine-based, which differs from that of other DPP-4 inhibitors. Linagliptin has pharmacokinetic properties that confer a prolonged terminal half-life (t_1/2 _> 100 h), and potent and durable DPP-4 inhibition (maximal inhibition of > 90% and inhibition 24 h after dosing of ~ 85% with linagliptin 5 mg at steady state); and unlike other DPP-4 inhibitors, it is primarily excreted via bile and the gut [13-15]. In Phase 3 trials, linagliptin has demonstrated clinically meaningful glycaemic efficacy and favourable safety/tolerability compared with placebo as monotherapy or in combination with metformin, metformin plus sulphonylurea, or pioglitazone [16-19].

To thoroughly determine the CV safety of linagliptin, we undertook a meta-analysis of the CV risk associated with linagliptin versus placebo or active comparators in patients with T2DM participating in the linagliptin Phase 3 study programme. This was a pre-specified meta-analysis in which suspected CV events were prospectively captured and adjudicated in a blinded fashion by an independent CV expert committee.

## Methods

### Study selection

The current meta-analysis included all randomized, double-blind, placebo- or active-controlled Phase 3 trials of linagliptin of > 12 weeks duration for which the database lock for the interim or final analyses was completed on or before 16 February 2010. This included eight studies that assessed linagliptin 5 mg or 10 mg/day versus placebo, glimepiride 1-4 mg/day, or voglibose 0.6 mg/day over 18-52 weeks as monotherapy or in combination with various common background therapies (for further details see Additional file 1, or the individual study publications [16-19]).

All patients from each study provided written informed consent. Local ethics committees/institutional review boards reviewed and approved all study protocols. All studies were conducted within ethical standards and in accordance with the Declaration of Helsinki and any applicable regulatory requirements.

### Analysis population

Common criteria across the Phase 3 trials included a diagnosis of inadequately controlled T2DM, age ≥ 18 years and, in most studies, body mass index (BMI) ≤ 40 kg/m^2^. Background medication with metformin was mandatory except where inclusion criteria required treatment-naïve patients, metformin-ineligible patients, or washout of pre-existing oral glucose-lowering drugs (including metformin). In all studies, rescue medication was provided with pioglitazone and/or insulin dose adjustment or supplementation for glycaemic deterioration, triggered by plasma glucose levels measurement on two separate occasions of > 13.3, > 11.1, or > 10.0 mmol/L after overnight fasts during the first 12, 12-24, or > 24 weeks, respectively.

### CV event data collection and adjudication

Adverse events (AEs) were captured and collected on-site by the study investigators using electronic case report forms. AEs were then mapped to preferred terms according to the Medical Dictionary for Regulatory Activities (MedDRA). A pre-specified list of trigger events (the Standard MedDRA Queries for ischaemic heart disease and cerebrovascular disorders) and all fatal events were identified for adjudication. In patients with a trigger event, an individual patient data package (patient profile and all available cardiology or neurology tests, laboratory tests, and medical records) was prepared for the adjudication committee. A cardiology or neurology clinical event committee, based on the data package, without knowledge of the treatment allocation, adjudicated on the trigger event and recorded the type of event as appropriate. These adjudicated events were collected and included in the clinical trial database upon completion of database lock for the full or interim analyses.

### Study endpoints

The primary endpoint was a composite of CV death (including fatal stroke and fatal myocardial infarction [MI]), non-fatal stroke, non-fatal MI, and hospitalization for unstable angina pectoris (UAP). Secondary endpoints were composites of: (i) CV death, non-fatal stroke, and non-fatal MI; (ii) all adjudicated CV events which included CV death, non-fatal stroke, non-fatal MI, UAP with or without hospitalization, stable angina pectoris (SAP), and transient ischaemic attacks (TIA); and (iii) FDA-defined custom major adverse CV events (MACE) derived from 34 unadjudicated MedDRA preferred terms for stroke and MI. Tertiary endpoints were the individual adjudicated components (as listed above) and total mortality.

### Statistical analysis

Analyses were based on individual patient data in the treated set, which was defined as all patients who were randomized and received at least one dose of study medication, in all Phase 3 studies. Descriptive statistics (incidence and incidence rates per 1000 patient-years) were determined for all endpoints in each of the pooled treatment groups within the treated set.

The primary analyses assessed the CV risk for all primary, secondary, and tertiary endpoints associated with linagliptin versus total comparators. Risk estimates were calculated using several common statistical methods that included: (i) the hazard ratio (HR) for time to first event calculated using the Cox proportional hazards model with adjustments for study and treatment group; (ii) the incidence-rate risk ratio (RR) for time to first event calculated using Poisson regression with adjustment for study and treatment group; (iii) the odds ratio (OR) for occurrence of events calculated using a stratified Exact test; and (iv) the RR for occurrence of events calculated using a stratified Cochran-Mantel-Haenszel (CMH) test with continuity correction for trials with zero events.

Sensitivity analyses included evaluation of the primary endpoint associated with linagliptin versus total comparators in pre-specified subgroups based on age, gender, race, and use of rescue medication, as well as further exploratory subgroups based on occurrence of hypoglycaemia and Framingham 10-year CV risk score. In addition, a *post hoc *analysis of the primary endpoint assessed events in linagliptin- and placebo-treated patients, taken only from placebo-controlled trials and placebo-controlled periods within trials. In addition, the influence on the primary endpoint of the factors: study, treatment, gender, race and time since diagnosis of diabetes, was investigated using Cox regression.

This combined study analysis was developed to fully adhere to the recent FDA guidance on assessment of CV safety for the development of oral glucose-lowering drugs [11].

## Results

### Patient characteristics and drug exposure

The current analysis included eight trials with a total of 5239 treated patients: 3319 received linagliptin once daily (5 mg: *n *= 3159, 10 mg: *n *= 160) and 1920 comparators (placebo: *n *= 977, glimepiride: *n *= 781, and voglibose: *n *= 162) (see Additional file 1). Patients were followed for a median (min, max) period of 175 (1, 617) days for linagliptin and 179 (1, 619) days for total comparators (169 [1, 367] for placebo and 409 [3, 619] for active comparators). Cumulative exposure (patient-years) was 2060 for linagliptin and 1372 for total comparators (422 for placebo, 872 for glimepiride, and 78 for voglibose).

The overall mean (± SD) age, BMI, and HbA1c were 58 ± 10 years, 29 ± 5 kg/m^2^, and 64 ± 10 mmol/mol (8.0 ± 0.9%), respectively, and 52.4% of patients had known T2DM for > 5 years. The predominant race was white (60.5%), and there were more males (55.5%) than females. In total, 60.8% of patients had metabolic syndrome (based on the International Diabetes Federation definition), 10.6% coronary artery disease, 2.5% cerebrovascular disease, and 3.3% peripheral artery disease. In addition, 64.6% of patients were hypertensive, and 38.3% were current or ex-smokers. The prevalence of some degree of renal impairment was 24.2% or 44.6% of all patients, according to the Cockcroft-Gault (CG) or Modification of Diet in Renal Diseases (MDRD) formulae, respectively. In general, these baseline demographics and clinical characteristics were comparable between the linagliptin and comparator groups (Tables 1 and 2). Table 3 shows changes from baseline to last measurement for HbA1c, systolic and diastolic blood pressure (BP), total cholesterol, triglycerides and body weight for the pooled linagliptin and the pooled comparator group. Changes were of similar magnitude in both groups for all parameters except for HbA1c, where a meaningful HbA1c reduction was seen for linagliptin, and body weight, where a modest weight increase was seen in the total comparator group. These findings were expected since linagliptin is a glucose-lowering drug and were provided to all patients in the linagliptin group, whereas ~50% of patients in the comparator cohort received placebo.

**Table 1:** Baseline demographics and clinical characteristics of the pooled cohorts from 8 trials of linagliptin versus total comparators (placebo and active treatment)

	Linagliptin (*n *= 3319)	Total comparators (*n *= 1920)
Gender, % of patients		
Male/female	53.7/46.3	58.6/41.4
Age, years	58 ± 10	58 ± 10
BMI, kg/m^2^	28.8 ± 5.0	29.1 ± 4.9
Race, % of patients		
White	59.7	61.8
Black	1.4	1.6
Asian	38.9	36.6
HbA_1c_, mmol/mol	65 ± 10	64 ± 10
HbA1c, %	8.1 ± 0.9	8.0 ± 0.9
FPG, mmol/L	9.3 ± 2.4	9.4 ± 2.3
Diabetes duration (known), % of patients		
≤ 1 years	12.1	12.2
1-5 years	34.8	36.6
> 5 years	53.1	51.2
Previous oral glucose-lowering agents, % of patients		
None	17.3	17.3
1	42.8	49.9
2	39.7	32.5
≥ 3	0.2	0.2
CV risk factors, % of patients		
Metabolic syndrome*	60.3	61.7
Coronary artery disease	10.4	11.0
Cerebrovascular disease	2.9	3.9
Peripheral artery disease	2.3	3.0
Hypertension	63.8	66.0
Ex-/current smoker	22.9/14.4	24.2/15.9
eGFR using CG/MDRD formulae, % of patients		
Normal	74.9/55.4	77.3/55.4
Mildly impaired	19.9/37.3	18.5/38.1
Moderately impaired	2.2/4.3	1.9/4.3
Severely impaired	0.1/0.1	0.1/0.1
CV medication, % of patients		
Acetyl-salicylic acid	29.5	30.5
Antihypertensive	60.0	63.0
Lipid-lowering therapy	39.5	42.1
Any of the above	72.8	75.5
Framingham 10-year CV risk score		
Score, %	9.8 ± 8.2	10.3 ± 8.4
Score > 15%, % of patients	27.8	31.1

*International Diabetes Federation definition. Values are mean ± SD, unless otherwise stated. BMI, body mass index; CG, Cockcroft-Gault; CV, cardiovascular; eGFR, estimated glomerular filtration rate; FPG, fasting plasma glucose; MDRD, Modification of Diet in Renal Disease study.

**Table 2:** Baseline demographics and clinical characteristics for the subset cohorts of patients participating in either placebo controlled trials or active controlled trials

	Active controlled trials (n = 2)		Placebo controlled trials (n = 6)	
	
	Linagliptin (*n *= 1097)	Active comparators* (*n *= 943)	Linagliptin (*n *= 2541)^†^	Placebo (*n *= 977)
Gender, % of patients,				
Male/female	62.4/37.6	62.8/37.2	51.9/48.1	54.7/45.3
Age, years	60 ± 10	60 ± 10	58 ± 10	57 ± 10
BMI, kg/m^2^	28.6 ± 5.1	29.5 ± 4.8	28.4 ± 5,0	28.7 ± 5.0
Race, % of patients				
White	60.2	69.9	52,0	54.0
Black	1.8	1.9	1.0	1.3
Asian	38.0	28.2	47.0	44.6
HbA_1c_, mmol/mol	62 ± 9	62 ± 10	66 ± 9	66 ± 10
HbA1c, %	7.8 ± 0.8	7.8 ± 0.9	8.2 ± 0.8	8.2 ± 0.9
FPG, mmol/L	9.1 ± 2.2	9.2 ± 2.3	9.4 ± 2.3	9.5 ± 2.3
Diabetes duration (known), % of patients				
≤ 1 years	8.1	8.4	13.8	16.0
1-5 years	40.6	38.8	32.7	34.4
> 5 years	51.3	52.8	53.6	49.6
Ex-/current smoker	29.6/17.6	29.5/15.7	21.1/14.7	19.1/16.1
eGFR using CG/MDRD formulae, % of patients				
Normal	76.2/59.3	78.0/52.3	73.4/57.3	76.7/58.3
Mildly impaired	20.6/35.5	18.7/41.4	20.8/35.3	18.3/34.9
Moderately impaired	1.6/3.7	1.1/4.1	2.6/4.3	2.7/4.5
Severely impaired	0/0	0/0	0.1/0.1	0.2/0.1
Framingham 10-year CV risk score				
Score, %	11.5 ± 8.1	11.6 ± 8.6	9.2 ± 8.0	9.1 ± 8.1
Score > 15%, % of patients	34.9	37.8	25.3	24.7

*Glimepiride (*n *= 781), voglibose (*n *= 162), ^**†**^One phase 3 trial was both placebo controlled (for the first 12 weeks) and actively controlled (for the first 26 weeks) and therefore the included 319 linagliptin patients are presented in both parts of the table. Values are mean ± SD, unless otherwise stated. BMI, body mass index; CG, Cockcroft-Gault; CV, cardiovascular; eGFR, estimated glomerular filtration rate; FPG, fasting plasma glucose; MDRD, Modification of Diet in Renal Disease study.

**Table 3:** Changes in CV risk factors from baseline to last measurement in the pooled cohorts of 8 trials of linagliptin versus total comparators (placebo and active treatment)

	Pooled linagliptin		Pooled total comparators	
	
	Baseline	Study end	Baseline	Study end
HbA_1c _, mmol/mol	64 ± 10	58 ± 11	63 ± 10	60 ± 13
HbA1c, %	8.1 ± 0.9	7.5 ± 1.0	8.0 ± 0.9	7.7 ± 1.2
Systolic BP, mmHg	131 ± 15	130 ± 15	132 ± 15	132 ± 15
Diastolic BP, mmHg	79 ± 9	78 ± 9	79 ± 9	79 ± 9
Total cholesterol, mmol/L	4.6 ± 0.6	4.6 ± 0.6	4.6 ± 0.6	4.7 ± 0.6
Triglycerides, mmol/L	2.6 ± 1.8	2.5 ± 1.8	2.6 ± 1.9	2.6 ± 1.9
Body weight, kg	78.9 ± 17.7	78.9 ± 17.6	81.0 ± 17.4	81.6 ± 17.9

Values are mean ± SD. BP, blood pressure.

### Adjudicated CV events

Table 4 summarizes the incidence of each CV endpoint. Overall, adjudicated primary CV events occurred in 11 (0.3%) patients receiving linagliptin and 23 (1.2%) receiving comparators (3 on placebo, 20 on glimepiride, and none on voglibose). Notably, the main contributor to the overall differences in incidence of the primary endpoint was the events in the head-to-head study of linagliptin versus glimepiride (mean ± SD dose of glimepiride at week 52: 3.0 ± 1.2 mg).

**Table 4:** Incidence and incidence rates of primary, secondary, and tertiary endpoints

	Linagliptin (*n *= 3319)		Active comparators* (*n *= 943)		Placebo (*n *= 977)		Total comparators (*n *= 1920)	
	Incidence *n *(%)	Incidence rate (per 1000 years)	Incidence *n *(%)	Incidence rate (per 1000 years)	Incidence *n *(%)	Incidence rate (per 1000 years)	Incidence *n *(%)	Incidence rate (per 1000 years)
Primary endpoints:								
CV death, stroke, MI, or UAP with hospitalization	11 (0.3)	5.3	20 (2.1)	21.2	3 (0.3)	7.0	23 (1.2)	16.8
Secondary endpoints:								
CV death, stroke, or MI	10 (0.3)	4.8	18 (1.9)	19.1	2 (0.2)	4.7	20 (1.0)	14.6
All major CV events	26 (0.8)	12.6	26 (2.8)	27.6	6 (0.6)	14.1	32 (1.7)	23.4
FDA-custom MACE	9 (0.3)	4.3	16 (1.7)	16.9	3 (0.3)	7.0	19 (1.0)	13.9
Tertiary endpoints:								
CV death	2 (0.06)	1.0	2 (0.2)	2.1	0	0	2 (0.1)	1.5
MI	6 (0.2)	2.9	6 (0.6)	6.3	1 (0.1)	2.3	7 (0.4)	5.1
Stroke	2 (0.06)	1.0	10 (1.1)	10.6	1 (0.1)	2.3	11 (0.6)	8.0
TIA	1 (0.03)	0.5	3 (0.3)	3.2	1 (0.1)	2.3	4 (0.2)	2.9
UAP with hospitalization	1 (0.03)	0.5	2 (0.2)	2.1	1 (0.1)	2.3	3 (0.2)	2.2
UAP without hospitalization	1 (0.03)	0.5	1 (0.1)	1.1	0	0	1 (0.05)	0.7
SAP	13 (0.4)	6.3	5 (0.5)	5.3	3 (0.3)	7.0	8 (0.4)	5.8
Total mortality	4 (0.1)	1.9	3 (0.3)	3.2	0	0	3 (0.2)	2.2

*Glimepiride (*n *= 781), voglibose (*n *= 162).

CV, cardiovascular; FDA, Food and Drug Administration; MACE, major adverse CV events; MI, myocardial infarction; SAP, stable angina pectoris; TIA, transient ischaemic attack; UAP, unstable angina pectoris.

**Figure 1:**
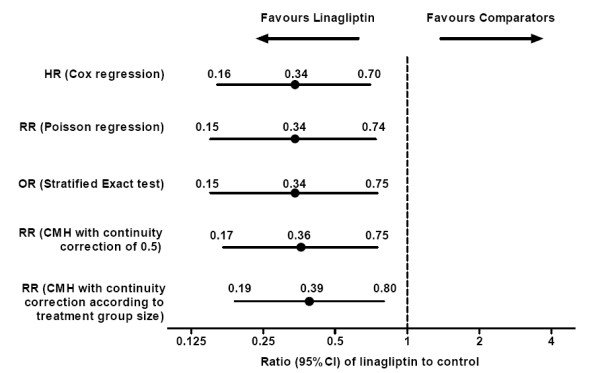
**Risk estimates for primary composite CV endpoint with linagliptin versus total comparators based on various statistical models**. CI, confidence interval; CMH, Cochran-Mantel-Haenszel; CV, cardiovascular; HR, hazard ratio; OR, odds ratio; RR, risk ratio.

**Figure 2:**
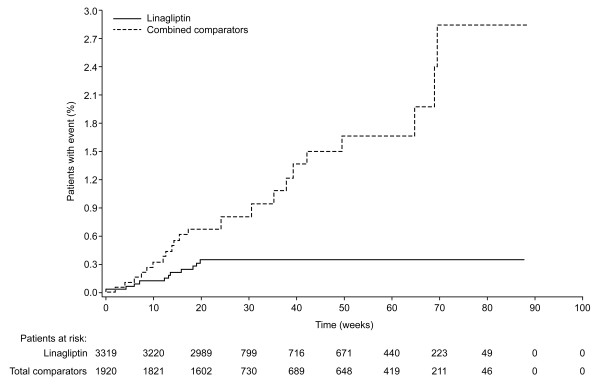
**Time to occurrence of primary composite CV event with linagliptin versus total comparator**.

Linagliptin treatment versus comparators was associated with reduced CV risk for the primary endpoint. The HR for the primary endpoint indicated a significant risk reduction, as did the OR and RR, for linagliptin versus comparator (i.e. upper bound of 2-sided 95% confidence interval [CI] < 1.0) (Figure 1). The difference in CV risk for the primary endpoint emerged after approximately 8 weeks and did not tend to plateau thereafter (Figure 2). Further analysis of the primary endpoint for linagliptin against placebo in those patients taken from the same placebo-controlled trials confirmed that linagliptin was associated with no significantly increased risk for the primary endpoint. The HR, OR, and RR with linagliptin versus placebo ranged from 0.69 to 0.90, but all had upper limits of 95% CIs that included 1.0.

The incidence rates for the primary endpoint and the associated CV risk reductions with linagliptin versus comparators in a number of subgroups (based on age, gender, race, rescue medication use, hypoglycaemia occurrence, or Framingham CV risk score) were generally consistent with the results in the overall population (Table 5 and Additional file 2). There were no significant increases in risk based on the HR and RR for the primary endpoint with linagliptin relative to comparators in any subgroup. However, linagliptin did achieve significant risk reductions over comparators in several subgroups, including males, whites, those receiving no rescue medication, those reporting no hypoglycaemia, and those with higher CV risk (Framingham CV risk score > 15%). Further, in the Cox regression analysis the HR was 0.36 (95% CI: 0.17-0.74) in a model where study, treatment, gender, race and time since diagnosis of diabetes were taken into account, i.e., fully in line with the HR seen in the simple Cox model (0.34).

**Table 5:** Subgroup analyses of primary endpoint for linagliptin versus total comparators based on Cox hazard model and CMH test

	Linagliptin, patients with events/total patients	Total comparators, patients with events/total patients	Cox HR (95% CI)	CMH RR (95% CI)
Age (years)*				
< 65	6/2390	9/1371	0.40 (0.14-1.14)	0.49 (0.19-1.27)
≥ 65	5/929	14/549	0.28 (0.10-0.79)	0.43 (0.18-1.03)
Gender				
Male	7/1782	20/1126	0.25 (0.10-0.60)	0.31 (0.13-0.71)
Female	4/1537	3/794	0.96 (0.21-4.37)	1.00 (0.32-3.17)
Race				
White	8/1981	22/1187	0.28 (0.12-0.63)	0.32 (0.15-0.70)
Black	0/46	0/31	n.a.	1.00 (0.29-3.50)
Asian	3/1292	1/702	1.63 (0.17-15.7)	1.20 (0.34-4.26)
Use of rescue medication				
No	8/3006	20/1666	0.29 (0.12-0.66)	0.37 (0.17-0.80)
Yes	3/313	3/254	0.80 (0.16-3.96)	0.91 (0.32-2.61)
Investigator-reported hypoglycaemia*				
No	11/3048	16/1604	0.39 (0.18-0.86)	0.45 (0.22-0.94)
Yes	0/271	7/316	n.a.	0.58 (0.13-2.58)
Framingham 10-year CV risk score				
≤ 15%	4/2395	5/1323	0.50 (0.13-1.90)	0.68 (0.24-1.95)
> 15%	7/923	18/597	0.31 (0.13-0.75)	0.40 (0.18-0.90)

*Exploratory analyses (all others pre-specified analyses).

CI, confidence interval; CMH, Cochran-Mantel-Haenszel; CV, cardiovascular; HR, hazard ratio; n.a., not applicable; RR, risk ratio.

**Figure 3:**
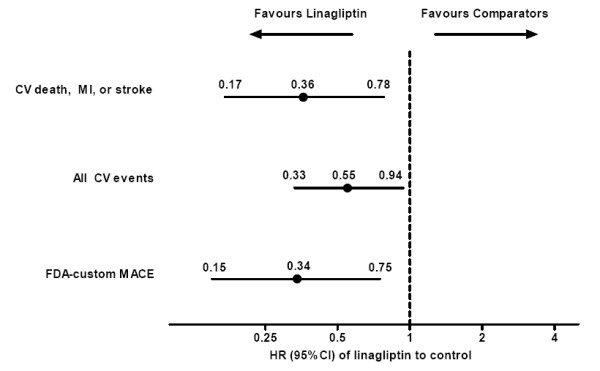
**HR estimates for secondary composite CV endpoints with linagliptin versus total comparators based on Cox hazard model**. CI, confidence interval; CV, cardiovascular; FDA, Food and Drug Administration; MACE, major adverse CV events; MI, myocardial infarction.

The HRs for all secondary endpoints indicated significantly lower CV risk with linagliptin than comparators (Figure 3). Similarly, significant ORs and RRs for linagliptin versus total comparators were also observed, with the single exception of the RR for all adjudicated CV events when evaluated with CMH method, where the upper 95% CI was equal to 1.0 (see Additional file 3). Of the tertiary endpoints, most HRs with linagliptin versus comparators showed either a favourable trend for risk reduction, as in the case of CV death, non-fatal MI, UAP, and TIA, or neutrality, as in the case of SAP and total deaths (Table 6); one exception was non-fatal stroke which was significantly lower with linagliptin versus comparators. Similar observations were made for OR and RR for linagliptin versus total comparators (see Additional file 3).

**Table 6:** Risk for tertiary individual CV endpoints with linagliptin versus total comparators based on Cox hazard model

	Cox HR (95% CI)
CV death	0.74 (0.10-5.33)
Non-fatal MI	0.52 (0.17-1.54)
Non-fatal stroke	0.11 (0.02-0.51)
TIA	0.17 (0.02-1.53)
UAP with hospitalization	0.24 (0.02-2.34)
UAP without hospitalization	0.73 (0.04-12.02)
SAP	1.06 (0.44-2.58)
Total mortality	1.02 (0.23-4.63)

CI, confidence interval; CV; cardiovascular; HR, hazard ratio; MI, myocardial infarction; SAP, stable angina pectoris; TIA, transient ischaemic attack; UAP, unstable angina pectoris.

## Discussion

This CV meta-analysis indicates that linagliptin may have a beneficial or neutral impact on CV outcomes in a large population of patients with T2DM compared with control treatments. Furthermore, the risk for CV events was unchanged, or lowered, across a number of pre-specified subgroups based on key demographic and clinical characteristics. These results include comparisons with placebo, as well as two active comparators, namely glimepiride (a second-generation sulphonylurea frequently used as second-line therapy in the USA and Europe) and voglibose (an α-glucosidase inhibitor commonly used in Asia), either as monotherapy or in combination with common oral glucose-lowering drugs.

The clinical characteristics of the overall study population were generally comparable to those reported in general T2DM populations. In this study, mean age was 58 years, BMI was 29 kg/m^2^, and 44.5% were females compared with age of 60 years, BMI of 32 kg/m^2^, and 52.4% females in the US National Health and Nutrition Examination Survey (NHANES) in 2003-2004 [20]. In this study versus the 2002 Cost of Diabetes in Europe (CODE)-2 study, the prevalence of previous MI was 10.6% versus 9.0% and of previous stroke was 2.5% versus 5% [21]. Furthermore, in the current study population, 44.6% had some degree of renal impairment versus 43.8% of those with self-reported T2DM in the NHANES population in 2009 (based on the MDRD equation) [22].

The incidence rates for CV events in this meta-analysis of linagliptin Phase 3 trials were relatively consistent with those observed in previous CV meta-analyses of other DPP-4 inhibitors' clinical trial programmes. The incidence rates (per 1000 patient-years) for the primary CV endpoint were 5.3 for linagliptin versus 16.8 for total comparators. In comparison, other CV meta-analyses reported incidence rates for custom MACE ranging from 5.8 to 14.6 with sitagliptin, saxagliptin, or vildagliptin and 9.0 to 14.1 with comparators [23-25]. Importantly, these CV meta-analyses have all reported relative risk for CV outcomes with DPP-4 inhibitors versus comparators that were below 1.0. However, not all of these risk estimates achieved statistical significance (based on upper bounds of 95% CI below 1.0). Risk reductions were significant in the present meta-analysis of linagliptin [HR 0.34 (95% CI 0.16-0.70)] and in the previous analysis of saxagliptin 2.5-10 mg [HR 0.43 (95% CI 0.23-0.80)] [23]. In contrast, risk estimates were non-significant for sitagliptin 100 mg [RR 0.68 (95% CI 0.41-1.12)] and vildagliptin 50 mg and 100 mg [RR 0.84 (95% CI 0.64-1.14) and 0.88 (95% CI 0.37-2.11)] [24,25].

Although the results of the different meta-analyses of DPP-4 inhibitors are not entirely comparable (due to differences in primary composite endpoints and CV adjudication methods), all are supportive of the hypothesis that, in general, DPP-4 inhibitor treatment does not have a deleterious impact on the incidence of CV events. The present analysis shows that linagliptin treatment does not increase CV risk and may even yield CV benefits in patients with T2DM. Meta-analyses of other DPP-4 inhibitors were frequently retrospective in nature. However, the pre-specified design of the present meta-analysis involved prospective and blinded adjudication of CV events, which should strengthen the validity of the current findings. In addition, this meta-analysis was based on individual patient data from a consistently designed, large clinical development programme; this allows consistent derivation of endpoints and extensive subgroup analyses and minimizes between-study heterogeneity that can confound analyses of unrelated studies.

There are several mechanisms that could underlie potential CV benefits for linagliptin. First, linagliptin may confer the beneficial effects of improved glycaemic control, including the lowering of postprandial glucose, without the potentially harmful effects of weight gain or increased hypoglycaemia [12,26]. Second, linagliptin increases GLP-1 and GIP levels which may provide beneficial cardioprotection; experimental and clinical data suggest that GLP-1 elevation can positively modulate lipid metabolism [26], reduce infarct size and improve cardiac function [26,27]. Third, DPP-4 substrates include not only incretins but also vasoactive peptides involved in inflammation, immunity, and CV function; some evidence, mainly from preclinical studies, indicates that reduced DPP-4 activity can lessen inflammation, stimulate endothelial repair, and blunt ischaemic injury [28]. Finally, linagliptin holds inherent anti-oxidative properties, most likely due to its xanthine-based molecular structure [29]. These properties, both directly through reduction of reactive oxygen species and indirectly through beneficial effects on inflammatory mediators and endothelial function, could reduce the atherosclerotic burden [30].

This analysis has several potential limitations. First, despite a large total patient exposure of 3432 years, individual patient exposure was of a maximum duration of 1.7 years; further longer-term data are needed to confirm the current findings. Second, the low incidence of CV events, low rates of triple oral therapy, and lack of insulin treatment all suggest that a large proportion of patients had less advanced T2DM, and thus a lower CV risk than those with more advanced T2DM. However, around 30% of patients had a baseline Framingham 10-year CV risk score of > 15% and more than a half also had > 5 years' known disease duration, which indicates a proportion of the population were at increased CV risk. Finally, the observed CV risk reductions for the primary and secondary endpoints were influenced by the differences in CV events in one study with linagliptin versus glimepiride. Despite this, it is important to note that glimepiride is an established and recommended second-line therapy with a well-characterized safety profile, which has not been directly linked to increased CV risk either as part of intensive treatment regimens or when compared with other conventional treatments [31]. Moreover, analysis of the pooled placebo studies alone confirmed that linagliptin did not increase CV risk against placebo.

## Conclusions

In summary, this pre-specified CV meta-analysis of a large Phase 3 programme that involved prospective and independent adjudication of CV events provides valuable new insights on the CV safety profile of linagliptin. Although a meta-analysis, with distinct limitations, the data indicate that linagliptin does not increase CV risk and, moreover, support a potential reduction of CV events with linagliptin compared with pooled comparators. These results suggest that linagliptin may be a valuable new therapeutic option for improving glycaemic control in patients with T2DM. The hypothesis that linagliptin may have CV benefits is currently being tested prospectively in the CAROLINA study (NCT01243424), the first large outcomes study to directly compare a DPP-4 inhibitor versus a sulphonylurea (glimepiride), predominantly as second-line therapy (i.e. on a background of metformin).

## List of abbreviations

AE: adverse event; BMI: body mass index; BP: Blood Pressure; CG: Cockcroft-Gault; CI: confidence interval; CMH: Cochran-Mantel-Haenszel; CV: cardiovascular; DPP-4: dipeptidyl peptidase-4; FDA: Food and Drug Administration; GIP: glucose-dependent insulinotropic polypeptide; GLP-1: glucagon-like peptide-1; HR: hazard ratio; MACE: major adverse CV events; MDRD: Modification of Diet in Renal Disease; MI: myocardial infarction; NHANES: National Health and Nutrition Examination Survey; OR: odds ratio; RR: risk ratio; SAP: stable angina pectoris; TIA: transient ischaemic attack; T2DM: type 2 diabetes mellitus; UAP: unstable angina pectoris.

## Competing interests

All authors are employees of Boehringer Ingelheim, the sponsor of the study.

## Authors' contributions

All authors meet criteria for authorship as recommended by the International Committee of Medical Journal Editors. All authors contributed to or participated in the design of the study, the analysis of data, the collection of data, and the writing or revision of the manuscript. All authors reviewed and approved the final version of the manuscript.

## Supplementary Material

Additional file 1 **Table S1 Overview of linagliptin Phase 3 clinical trials included in the CV meta-analysis**. Click here for file

Additional file 2 **Figure S1 Subgroup analyses of incidence rates of primary endpoint for linagliptin versus total comparators**. Click here for file

Additional file 3 **Table S2 Risk for other CV endpoints with linagliptin versus total comparators based on various statistical methods**. Click here for file
