# Supplementary material for: Cardiovascular safety with linagliptin in patients with type 2 diabetes mellitus: a pre-specified, prospective, and adjudicated meta-analysis of a phase 3 programme
Source: Cardiovasc Diabetol. 2012 Jan 10;11:3. doi: 10.1186/1475-2840-11-3 (PMC3286367; doi:10.1186/1475-2840-11-3)
Supplement: Additional file 1 — Table S1 Overview of linagliptin Phase 3 clinical trials included in the CV meta-analysis. [file 1475-2840-11-3-S1.PDF]

Table S1 Overview of linagliptin Phase 3 clinical trials included in the CV meta-analysis

Study	Treatment	Patients	Background	Follow-up (weeks)	Reference/ NCT #
1218.15	Linagliptin 5 mg<br>Placebo	259<br>130	Pioglitazone	24	Gomis *et al* [18]<br>NCT00641043
1218.16	Linagliptin 5 mg<br>Placebo	336<br>167	None	24	Del Prato *et al* [16]<br>NCT00621140
1218.17	Linagliptin 5 mg<br>Placebo	523<br>177	Metformin	24	Taskinen *et al* [17]<br>NCT00601250
1218.18	Linagliptin 5 mg<br>Placebo	792<br>263	Metformin + sulphonylurea	24	Owens *et al* [19]<br>NCT00602472
1218.20	Linagliptin 5 mg<br>Glimepiride 1–4 mg	778<br>781	Metformin	52*	NCT00622284
1218.23	Linagliptin 5 mg<br>Linagliptin 10 mg<br>Voglibose 0.6 mg<br>Placebo	159<br>160<br>162<br>80	None	26	NCT00654381
1218.35	Linagliptin 5 mg<br>Placebo	161<br>84	Sulphonylurea	18	NCT00819091
1218.50	Linagliptin 5 mg<br>Placebo†	151<br>76	None	18†	NCT00740051

*Interim analysis at database lock; study continued for further 52 weeks.
†Placebo only during 18 weeks; switched to glimepiride for further 34 weeks (in metformin ineligible patients).
