# Supplementary material for: Cardiovascular safety with linagliptin in patients with type 2 diabetes mellitus: a pre-specified, prospective, and adjudicated meta-analysis of a phase 3 programme
Source: Cardiovasc Diabetol. 2012 Jan 10;11:3. doi: 10.1186/1475-2840-11-3 (PMC3286367; doi:10.1186/1475-2840-11-3)

Figure S1 Subgroup analyses of incidence rates of primary endpoint for linagliptin versus total comparators.

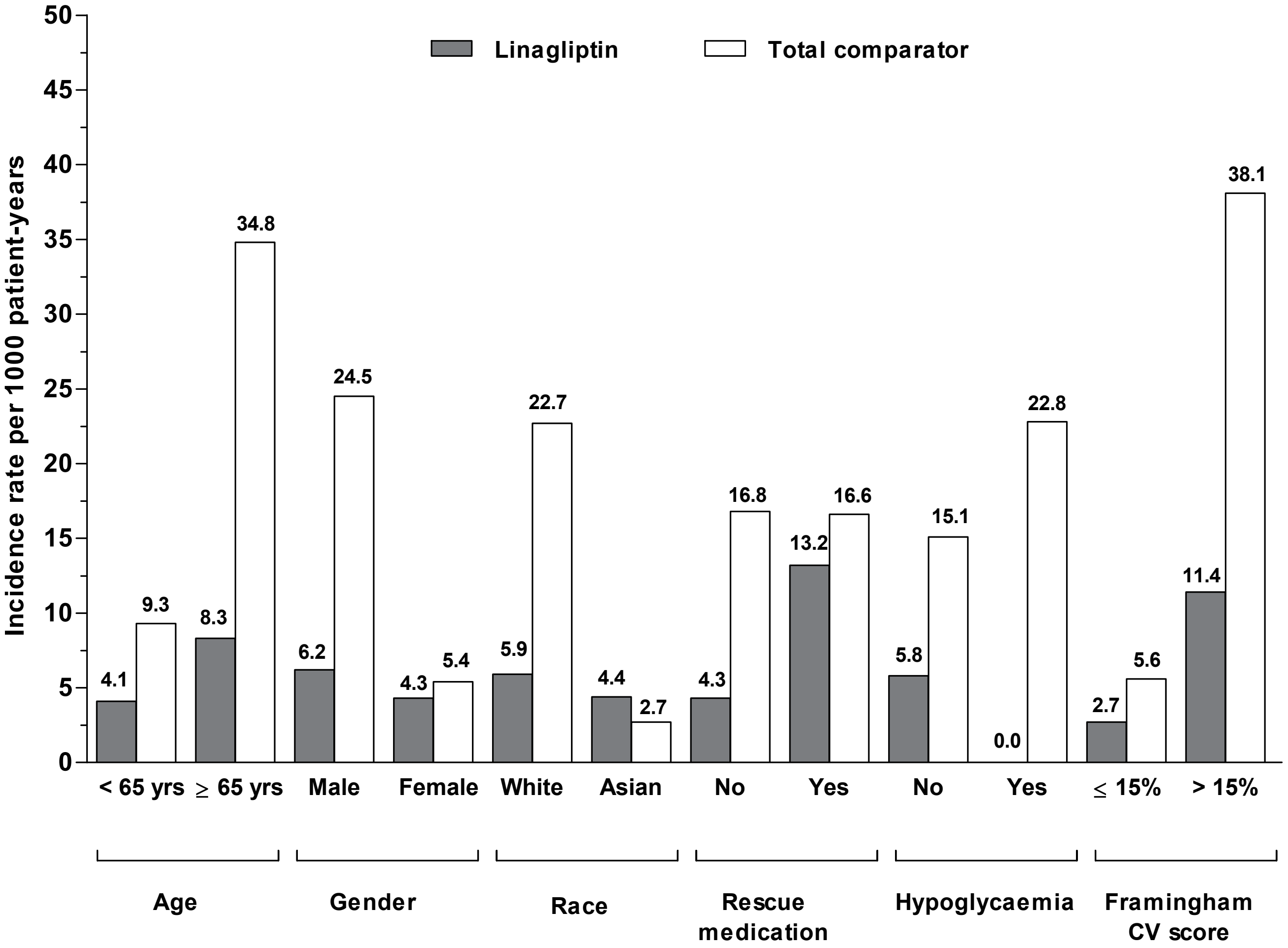

Supplement: Additional file 2 — Figure S1 Subgroup analyses of incidence rates of primary endpoint for linagliptin versus total comparators. [file 1475-2840-11-3-S2.PDF]
