# Supplementary material for: Cardiovascular safety with linagliptin in patients with type 2 diabetes mellitus: a pre-specified, prospective, and adjudicated meta-analysis of a phase 3 programme
Source: Cardiovasc Diabetol. 2012 Jan 10;11:3. doi: 10.1186/1475-2840-11-3 (PMC3286367; doi:10.1186/1475-2840-11-3)
Supplement: Additional file 3 — Table S2 Risk for other CV endpoints with linagliptin versus total comparators based on various statistical methods. [file 1475-2840-11-3-S3.PDF]

**Table S2 Risk for other CV endpoints with linagliptin versus total comparators based on various statistical methods**

| | Poisson RR (95% CI) | OR (95% CI) | CMH RR (95% CI) |
|---|---|---|---|
| Secondary composite CV endpoints | | | |
| CV death, non-fatal MI or non-fatal stroke | 0.37 (0.15–0.84) | 0.37 (0.15–0.84) | 0.42 (0.20–0.90) |
| All adjudicated CV events | 0.56 (0.32–0.98) | 0.56 (0.32–0.99) | 0.59 (0.35–1.00) |
| FDA-custom MACE | 0.34 (0.13–0.81) | 0.35 (0.14–0.82) | 0.39 (0.18–0.85) |
| Tertiary individual CV endpoints | | | |
| CV death | 0.67 (0.05–9.18) | 0.58 (0.04–7.98) | 0.94 (0.26–3.34) |
| Non-fatal MI | 0.57 (0.16–1.98) | 0.50 (0.14–1.72) | 0.70 (0.26–1.87) |
| Non-fatal stroke | 0.12 (0.01–0.55) | 0.10 (0.01–0.48) | 0.34 (0.12–0.91) |
| TIA | 0.17 (0.00–1.68) | 0.14 (0.00–1.46) | 0.54 (0.17–1.72) |
| UAP with hospitalization | 0.22 (0.00–2.76) | 0.19 (0.00–2.40) | 0.60 (0.18–1.95) |
| UAP without hospitalization | 0.66 (0.01–52.18) | 0.58 (0.01–45.42) | 0.89 (0.23–3.47) |
| SAP | 1.08 (0.42–3.01) | 0.94 (0.36–2.62) | 1.06 (0.49–2.29) |
| Total mortality | 0.89 (0.15–6.05) | 0.77 (0.13–5.27) | 1.06 (0.34–3.29) |

CI, confidence interval; CMH, Cochran–Mantel–Haenszel; CV, cardiovascular; FDA, Food and Drug Administration; MACE, major adverse CV events; MI, myocardial infarction; OR, odds ratio; RR, risk ratio; SAP, stable angina pectoris; TIA, transient ischaemic attack; UAP, unstable angina pectoris.
